# Draft Whole-Genome Sequence of *Morganella morganii* Serotype O:1ab

**DOI:** 10.1128/genomeA.00453-15

**Published:** 2015-05-21

**Authors:** John H. E. Nash, N. Martin Young

**Affiliations:** a Laboratory for Foodborne Zoonoses, Public Health Agency of Canada, Guelph, Ontario, Canada; b Human Health Therapeutics Portfolio, National Research Council of Canada, Ottawa, Ontario, Canada

## Abstract

*Morganella morganii* is a facultative pathogen of humans, causing urinary tract and postsurgical infections. Here, we report a high-quality draft assembly of the O:1ab serotype.

## GENOME ANNOUNCEMENT

*Morganella morganii* is a facultative anaerobic Gram-negative bacterium usually prevalent as a human commensal organism. However, it can act as an opportunistic pathogen, causing postsurgical and urinary tract infections (1–4). It is a member of the *Proteeae* tribe of the *Enterobacteriaceae* family, and it is related to the pathogens *Proteus mirabilis* and *Providencia alcalifaciens* (5). The structure of the core region of the lipopolysaccharide from the *M. morganii* O1 type strain ATCC 49993 was recently determined, along with its associated genomic loci (6), and we report its genome sequence here.

Genomic DNA was extracted using the Qiagen EZ1 DNA tissue kit (catalog no. 953034). It was sequenced using the 454-GS Titanium sequencing platform (Roche Diagnostics Corp., Indianapolis, IN) at McGill University and the Génome Québec Innovation Centre (Montreal, Canada) to approximately 40-fold sequence coverage. High-quality draft genome assemblies were created using the MIRA assembler, version 4.0.2 (7), and the assembly was proofread using the Gap5 program from the Staden package (8).

The draft assembly was annotated using the Prokka annotation package, version 1.11 (9). Note that a draft annotation of this genome assembly was used to identify the lipopolysaccharide (LPS) outer core locus (6). A subsequent reannotation of the genome led to renumbering of the loci described in Table 3 in a study by Vinogradov et al. (6), such that each gene number has been incremented by 1, i.e., the former Mm0Y_02796 is now Mm0Y_02797, etc.

The draft whole-genome assembly contains 3,955,391 nucleotides (nt) represented in 61 contigs, with 13 >100,000 nt in size, with the largest containing 715,244 nt. The G+C content is 52.49%, and 3,795 proteins were identified. Although our primary reason for sequencing the genome of strain 8066 was to identify candidates for genes controlling the synthesis of the LPS, we note that this strain also contains several genes associated with resistance to multiple antibiotics and heavy metals. This is consistent with other reports of the presence of antimicrobial resistance and other virulence factors in *M. morganii* (10, 11). Therefore, we have made the raw sequence reads available for further study by the scientific community by depositing them in the NCBI Sequence Read Archive.

### Nucleotide sequence accession numbers. 

The whole-genome shotgun project of *M. morganii* ATCC 49993 has been deposited at DDBJ/EMBL/GenBank under the accession no. LAGC00000000. The version described in this paper is version LAGC01000000. The raw sequence reads used to generate this draft whole-genome assembly are available from the NCBI Sequence Read Archive under accession no. SRR1919351.
